# Does *Saccharomyces cerevisiae* Require Specific Post-Translational Silencing against Leaky Translation of Hac1up?

**DOI:** 10.3390/microorganisms9030620

**Published:** 2021-03-17

**Authors:** Ali Tehfe, Talia Roseshter, Yulong Wei, Xuhua Xia

**Affiliations:** 1 Department of Biology, University of Ottawa, 30 Marie Curie, P.O. Box 450, Station A, Ottawa, ON K1N 6N5, Canada; atehf102@uottawa.ca (A.T.); trose017@uottawa.ca (T.R.); ywei025@uottawa.ca (Y.W.); 2 Ottawa Institute of Systems Biology, University of Ottawa, Ottawa, ON K1H 8M5, Canada

**Keywords:** *HAC1*, non-spliceosome splicing, unfolded protein response, degron

## Abstract

*HAC1* encodes a key transcription factor that transmits the unfolded protein response (UPR) from the endoplasmic reticulum (ER) to the nucleus and regulates downstream UPR genes in *Saccharomyces cerevisiae*. In response to the accumulation of unfolded proteins in the ER, Ire1p oligomers splice *HAC1* pre-mRNA (*HAC1*^u^) via a non-conventional process and allow the spliced *HAC1* (*HAC1^i^*) to be translated efficiently. However, leaky splicing and translation of *HAC1*^u^ may occur in non-UPR cells to induce undesirable UPR. To control accidental UPR activation, multiple fail-safe mechanisms have been proposed to prevent leaky *HAC1* splicing and translation and to facilitate rapid degradation of translated Hac1^u^p and Hac1^i^p. Among proposed regulatory mechanisms is a degron sequence encoded at the 5′ end of the *HAC1* intron that silences Hac1^u^p expression. To investigate the necessity of an intron-encoded degron sequence that specifically targets Hac1^u^p for degradation, we employed publicly available transcriptomic data to quantify leaky *HAC1* splicing and translation in UPR-induced and non-UPR cells. As expected, we found that *HAC1^u^* is only efficiently spliced into *HAC1^i^* and efficiently translated into Hac1^i^p in UPR-induced cells. However, our analysis of ribosome profiling data confirmed frequent occurrence of leaky translation of *HAC1^u^* regardless of UPR induction, demonstrating the inability of translation fail-safe to completely inhibit Hac1^u^p production. Additionally, among 32 yeast *HAC1* surveyed, the degron sequence is highly conserved by *Saccharomyces* yeast but is poorly conserved by all other yeast species. Nevertheless, the degron sequence is the most conserved *HAC1* intron segment in yeasts. These results suggest that the degron sequence may indeed play an important role in mitigating the accumulation of Hac1^u^p to prevent accidental UPR activation in the *Saccharomyces* yeast.

## 1. Introduction

Folding of nascent proteins in the endoplasmic reticulum (ER) is an error-prone process in eukaryotic cells, and the accumulation of unfolded/misfolded proteins can lead to lethal consequences if left unregulated [1]. In response to the increase of unfolded/misfolded proteins, yeasts have evolved a complex Ire1p+Hac1p-mediated signaling pathway to tightly control protein folding [2,3]. This unfolded protein response (UPR) increases ER folding capacity by increasing the production of specific chaperon proteins, reducing folding load by reducing global translation activity and triggers apoptosis when UPR fails to restore ER homeostasis.

During ER stress, the ER transmembrane kinase/endonuclease Ire1p [4] detects UPR via a core luminal domain [5,6] and responds by undergoing trans-autophosphorylation and congregation into discrete foci of Ire1p oligomers [7]. *HAC1* pre-mRNA (*HAC1^u^*) is recruited, through recognition of its 3′ bipartite element (3′ BE) [8], to the Ire1p oligomers to undergo splicing. What is particular about *HAC1^u^* splicing is that it is not spliced through the conventional spliceosome mechanism.

**Figure 1:**
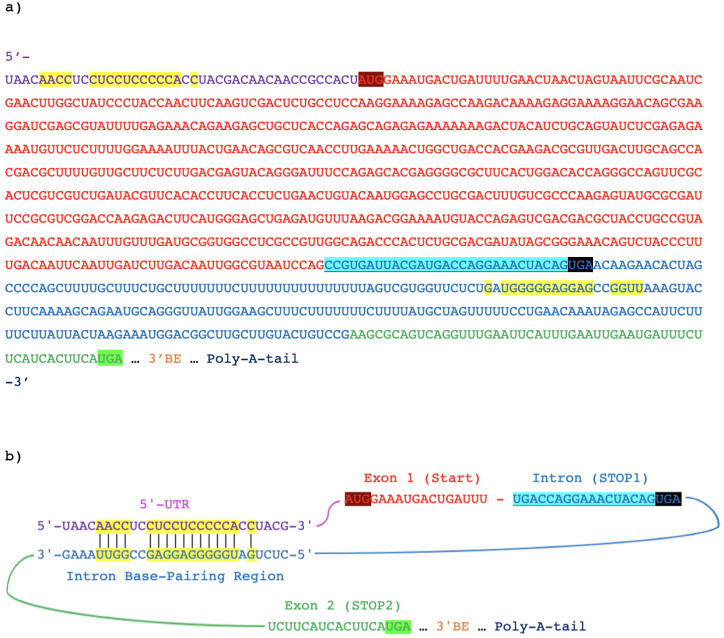
The *HAC1* gene in *S. cerevisiae*. (**a**) The annotated *HAC1*^u^ mRNA illustrating its sequence elements: 5′ untranslated region (5′ UTR) in purple, first exon in red, the intron in blue, and the second exon in green. The start codon (START) is highlighted in dark red, and the in-frame stop codons in the intron (STOP1) and in the second exon (STOP2) are respectively highlighted in black and green. Nucleotides involved in the long-ranged base-pair interactions between the 5′ UTR and intron are highlighted in yellow, while the intronic sequence proposed to encode a C-terminal degron in Hac1^u^p is shaded in blue. (**b**) An illustration showing the base-pairs formed between the 5′ UTR and intron in *HAC1*^u^ to inhibit translation initiation of the pre-mRNA, with the same color scheme as in (**a**).

To form mature *HAC1* mRNAs (*HAC1^i^*) in *Saccharomyces cerevisiae*, oligomerized Ire1p cleaves out a single 252nt *HAC1* intron (Figure 1a) [4,9] with its RNAse activity [5,7,9,10,11,12,13], and the exons are then ligated by the tRNA ligase Trl1p [14,15,16,17,18,19]. Aside from *S. cerevisiae*, Ire1p-mediated *HAC1* splicing has been observed in *Candida albicans* [20,21], *Candida glabrata* [21], *Kluyveromyces lactis* [21], *Pichia pastoris* [22], *Hansenula polymorpha* [23], and *Yarrowia lipolytica* [24] under ER stress conditions. Furthermore, through a comparative gene study, Hooks and Griffiths-Jones [25] determined *HAC1* homologs in 19 yeasts with intron lengths ranging from 19nt to 379nt, with most yeast lineages that diverged early having short *HAC1* introns.

The *HAC1^i^* mRNA is efficiently translated when the *HAC1* intron is removed through Ire1p-mediated splicing, and the translated protein (Hac1^i^p) is a key transcription activator that transmits UPR from the ER to the nucleus [4,9,26,27,28]. Hac1^i^p contains a nuclear localization signal that binds to UPR related genes such as *KAR2, PDI1, EUG1, FKB2,* and *LHS1* that feature one of the two conserved UPR elements (UPREs): UPRE1 (GACAGCGTGTC) [9,29,30,31], and UPRE2 (TACGTGT) [31,32,33,34]. This signaling pathway increases the production of UPR related proteins to stimulate protein folding [4,9,32]. 

In absence of UPR, the unspliced *HAC1^u^* mRNA is still constitutively transcribed and stable, and it is thought to be slowly translated into Hac1^u^p [4,9]. Hac1^u^p is a truncated protein because of the presence of an in-frame stop codon (STOP1) in the intronic sequence (Figure 1a) [35]. In *S. cerevisiae*, the truncated Hac1^u^p misses the 18 amino acids transcriptional activation domain [35] encoded by the second exon. Consequently, Hac1^u^p has reduced transactivation activity [4] on UPRE promoter elements [36] relative to Hac1^i^p. 

As a mechanism to block undesirable translation of *HAC1^u^*, part of the intron forms base pairs with the 5′ untranslated region (5′ UTR) in the unspliced form (Figure 1b). This interaction has been experimentally shown to inhibit the undesired translation of *HAC1^u^* pre-mRNAs [11,26,37,38,39,40] and may prevent unnecessary UPR activation in absence of ER stress [41]. Indeed, leaky translation of *HAC1^u^* was considered minimal given the well-documented intron-mediated translation inhibition (Figure 1b). Another fail-safe mechanism that prevents leaky splicing is handled by the Kar2p chaperon. When Kar2p is free from protein folding, it binds to Ire1p to hinder Ire1p oligomerization and prevent its RNase splicing activity [8,42,43]. A third fail-safe mechanism against undesired *HAC1^u^* splicing is the rapid removal of the 3′ BE in the 3′ UTR of *HAC1^u^* transcripts under non-UPR-induced (hereafter referred to as just non-UPR) condition. This prevents the translocation of *HAC1^u^* to Ire1 foci [11] and limits Ire1p-mediated splicing. Resultantly, *HAC1^i^* mRNAs are hardly detectable in non-UPR cells [44]. 

Nonetheless, leaky translation and leaky splicing of *HAC1^u^* may still occur during non-ER stress, and this is undesirable because it could lead to the accumulation of Hac1^i^p and accidental induction of UPR. While leaky translation of *HAC1^u^* may be rare [39], there is substantial evidence that Hac1^u^p will accumulate without efficient protein degradation [38] and may lead to accidental induction of UPR [41]. Thus, rapid degradation of Hac1p may be a much-required fail-safe mechanism in non-UPR cells to counteract leaky translation of Hac1^u^p and Hac1^i^p. It was previously proposed that this process requires a nuclear localization signal (29)RKRAKTK(35) that is encoded in the first exon [45] and is recognized by the ubiquitin-proteasome pathway [38,45]. Since the degradation signal is located in the first exon, it is shared by Hac1^u^p and Hac1^i^p, which explains why both protein forms are highly unstable in the yeast [26,46] with half-life of about 1.5–2.0 min [26,45,46]. 

Recently, Di Santo et al. [38] suggest that *S. cerevisiae* contains an additional post-translational regulatory signal, encoded by a 10 amino acid “degron” at the C-terminus of Hac1^u^p that is located just before STOP1 (Figure 1a). Their meticulous experimental design involves *GFP*-*HAC1* intron constructs in a fluorescent measurement-based assay. To summarize, they found that the degron plays a functionally important role in the post-translational silencing of Hac1^u^p. When the degron was present in the construct, fluorescence was barely detectable in cells expressing green fluorescent protein (GFP), regardless of the presence of the downstream intronic sequence. When the degron sequence was removed from the intron, fluorescence was restored to a level comparable with the intron-less constructs. Furthermore, GFP silencing was unaffected when the degron was recoded with synonymous codons, but silencing was abolished when the 10 amino acid sequence was altered or contained a premature stop codon. To further demonstrate that the degron acts by promoting Hac1^u^p degradation and not by disrupting translation, the *GFP*-*HAC1* intron construct were non-covalently linked by an upstream *HA-mRuby* reporter, which was expressed only if translation was not affected, and *HA-mRuby* gene expression was indeed not affected by the presence of the degron [38]. Together, these reporter constructs demonstrated that the translation of the C-terminus degron could effectively silence *HAC1^u^* expression by rapidly degrading Hac1^u^p. 

As mentioned previously, *HAC1* may encode a nuclear localization sequence in the first exon that signals for the rapid degradation of both Hac1^i^p and Hac1^u^p. In addition, translation of *HAC1^u^* is blocked by a 5′ UTR loop. Nonetheless, having an additional fail-safe degron that specifically targets Hac1^u^p for degradation would be advantageous if the *HAC1^u^* translation block does not adequately eliminate leaky *HAC1^u^* translation. Furthermore, if this intron-encoded degron is an important post-translational control, then we expect this sequence to be evolutionarily conserved among closely related yeast species under similar selection pressure.

Our investigation considered the *HAC1* gene sequence in 32 yeast species and the transcriptomics data from UPR-induced and non-UPR *S. cerevisiae* yeast. Transcript profiling analyses suggest that *HAC1^u^* is efficiently spliced into HAC1^i^, and *HAC1^i^* is efficiently translated into Hac1^i^p in UPR-induced cells. In contrast, *HAC1^u^* is constitutively translated into Hac1^u^p in both UPR-induced and non-UPR cells, albeit with many translational regulatory mechanisms featured in the Ire1p-Hac1p pathway. Thus, having an intron-encoded degron signal to specifically degrade undesired Hac1^u^p production would protect cells against accidental activation of UPR. Expectedly, we found that the intronic degron sequence upstream of STOP1 is highly conserved by *Saccharomyces* yeasts. However, conservation at putative degron sequences was poor in all non-*Saccharomyces* yeast species surveyed. Nevertheless, the first four degron-encoded amino acids are highly conserved, and the putative degron was the most conserved intronic region among yeast species. 

## 2. Materials and Methods

### 2.1. Retrieving the HAC1 Genes from 32 Yeast Species and Determining Their Introns

We retrieved the complete *HAC1* gene and 18S rRNA sequences of 32 yeast species belonging to the Saccharomycetales order. These species were selected because all have available *HAC1* and 18S rRNA gene records in the National Center for Biotechnology Information (NCBI) Gene Database. Of these species, 14 belong to the Saccharomycetaceae family and 18 belong to eight other families (Trichomonascaceae, Phaffomycetaceae, Ascoideaceae, Debaryomycetaceae, Metschnikowiaceae, Pichiaceae, Phaffomycetaceae, and Dipodascaceae). Furthermore, to show that the *IRE1* gene is present in each yeast species surveyed, the *IRE1* Gene ID was retrieved from NCBI Gene Database and listed in Supplemental File S1.

The *HAC1* 5′ and 3′ splice site information of *Saccharomyces cerevisiae*, *Candida orthopsilosis*, *Candida albicans*, *Lodderomyces elongisporus*, *Clavispora lusitaniae*, *Scheffersomyces stipitis*, *Meyerozyma guilliermondii*, and *Debaryomyces hansenii* were obtained from Iracane et al. [47] and those of *Yarrowia lipolytica*, *Candida glabrata*, *Lachancea thermotolerans*, *Eremothecium gossypii*, *Kluyveromyces lactis*, *Zygosaccharomyces rouxii*, *Saccharomyces paradoxus*, *Naumovozyma castellii*, and *Candida dubliniensis* were obtained from Hooks et al. [25]. Exon-intron junctions from these *HAC1* sequences, along with those of *Ogataea polymorpha* which were annotated in the National Center for Biotechnology Information (NCBI) database, were extracted (15nt from each side) and scored by position weight matrix (PWM) [48] in Data Analysis in Molecular Biology and Evolution (DAMBE7) [49] with default settings. The resulting PWM scores (Supplemental File S1) were used to characterize the *HAC1* 5′ and 3′ splice sites of the remaining 14 species which do not have fully annotated *HAC1* splice junctions in the NCBI GenBank. 

### 2.2. Determining the Putative 3′ Degron Sequence and Its Degree of Conservation in 32 Yeast Species

The putative degron sequence was previously determined in the budding yeast *S. cerevisiae* by Di Santo et al. [38], which consists of 29nt from the 5′ end of the intron sequence followed by the first in-frame stop codon (the stop codon UGA designated as STOP1 in Figure 1). The truncated *HAC1^u^* in *S. cerevisiae*, which is 687nt from the start codon in the first exon until STOP1, translates into a 228 amino acid-long truncated Hac1^u^p. To determine a putative degron sequence in the other 31 yeast species surveyed in this study, we manually identified the first in-frame stop codon (STOP1) in the intron of their *HAC1* genes such that the nucleotide sequence of the truncated *HAC1^u^*, from start codon until STOP1, is translatable (divisible by 3), and the putative degron was determined as the sequence from the beginning of the intron until STOP1. 

Most *HAC1* introns contained an in-frame stop codon (STOP1). However, *Candida dubliniensis*, *Candida albicans*, *Sugiyamaella lignohabitans*, *Ascoidea rubescens*, and *Yarrowia lipolytica* did not contain an in-frame STOP1 (Supplemental File S1). Additionally, *Cyberlindnerajadinii* and *Candida orthopsilosis* contain in-frame stop codons TGA and TAA, respectively, after just one in-frame codon in intron, and one *Candida haemulonii* has an in-frame stop codon TGA after just two in-frame codons in intron; the sequence between the 5′ end of intron to STOP1 in these species are too short to constitute degron sequences. In brief, a total of 24 out of 32 species contain an in-frame STOP1 and the lengths of the degron sequences (including STOP1) range between 17nt to 101nt, coding between five to 33 sense codons.

To determine the degree of conservation between degrons in 32 species, the degron segments were retrieved as nucleotide sequences and as translated amino acids. In *S. cerevisiae*, the degron-encoded codons consist of 29nt upstream of STOP1 plus 1nt at the 3′ end of first exon; similarly, up to 2nt from the 3′ end of exon 1 was considered by the first degron-encoded codon for select yeast species (See Supplemental File S1) to ensure the degron is translatable as an amino acid sequence. The nucleotide and amino acid degron sequences were then aligned by Multiple Alignment using Fast Fourier Transform (MAFFT) with the slow but accurate G-INS-i option (global alignment approach using the Needleman-Wunsch algorithm) [50] implemented in DAMBE. Next, a heatmap was generated for the alignments with the degron in *S. cerevisiae* as reference, and a total similarity score (indicating the total number of matching nucleotides and amino acids at the degron) was calculated for each species.

### 2.3. Reconstructing the Phylogenetic Relationship of 32 Yeast Species and Determining Relative Conservation at HAC1 Intronic and Exonic Regions

Two phylogenetic trees were constructed for the 32 yeast species, one with complete *HAC1* genes and the other with 18S rRNAs. Multiple sequence alignments (MSA) of *HAC1* and 18S rRNA nucleotide sequences were performed using Multiple Sequence Comparison by Log-Expectation (MUSCLE) [51] implemented in DAMBE. Then, phylogenetic relationships were inferred from aligned sequences using PHYML (a phylogeny software based on the maximum-likelihood principle) approach [52] with bootstrap = 500, tree improvement = Nearest Neighbour Interchange (NNI), and best model selected by Smart Model Selection (SMS) [53] = Generalised time reversible model (GTR + G + I) based on Akaike Information Criterion (AIC). Then, the phylogenetic trees were illustrated using the Interactive Tree of Life (iTOL) v4 [54]. 

Relative degrees of site-specific conservation among aligned *HAC1* nucleotide sequences were measured using the Phylogenetic Analysis with Space/Time models (PHAST) package [55]. The phylogenetic tree was fitted to the MSA by maximum likelihood using phyloFit, and the MSA and resulting neutral model file were used to score conservation with PhastCons. For both PHAST programs (phyloFit and PhastCons), parameters were left as default. PhastCons works by fitting a phylogenetic hidden Markov model (phylo-HMM) to the data by maximum likelihood, subject to constraints designed to calibrate the model across species groups, and then predicting and assigning log-odds scores to conserved elements based on this model [56]. Conservation scores are posterior probabilities generated at each site by their conserved state, and the score enables comparison of conservation across sites in a sequence alignment [56]. Relative conservation of the exonic and intronic (degron and downstream) regions was calculated as the average conservation score considering all nucleotide sites in the gene segment. To ensure that mean conservation results were not skewed (e.g., some exonic regions partially mapping to intronic regions of other sequences in the alignment), the process was repeated with coding DNA-sequence (CDS) and intronic *HAC1* sequences separately.

### 2.4. Retrieving and Processing S. cerevisiae Transcriptomic Data 

The experimental information (Supplemental File S1) and FASTQ (FASTA with quality score) transcriptomics data of *S. cerevisiae* from Van Dalfsen et al. [57] were retrieved from Gene Expression Omnibus (GEO) Datasets [58]. These include the datasets of untreated wild type cells (RNA-Seq: SRR7265163 and SRR7265164; Ribo-Seq: SRR7265151 and SRR7265152), cells treated with dithiothreitol (DTT) to induce UPR (RNA-Seq: SRR7265165 and SRR7265166; Ribo-Seq: SRR7265153 and SRR7265154), and cells treated with tunicamycin (TM) to induce UPR (RNA-Seq: SRR7265167 and SRR7265168; Ribo-Seq: SRR7265155 and SRR7265156).

**Figure 2:**
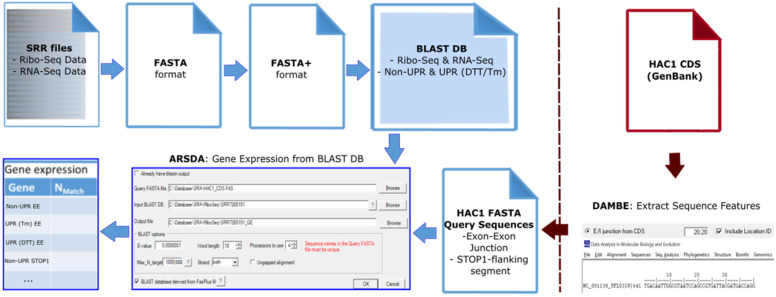
Processing of RNA-Seq and Ribo-Seq data. ARSDA [59] was used to convert FASTQ datasets into the BLAST database files for profiling analyses. The query sequences were made from the *HAC1* sequence file, retrieved from NCBI GenBank [60] using DAMBE7 [49].

Analyzing RNA-Seq Data (ARSDA) [59] was used to process the original FASTQ files in the following workflow: FASTQ → FASTA → FASTA+ → Basic Local Alignment Search Tool (BLAST) database (Figure 2). After initial conversion to FASTA format, the data was trimmed (‘FileǀRTrim Fasta Sequences’) to exclude the 3′-poly-A tails that were added to the Ribo-Seq sequences during library generation, with minimum length set to 50 and reads with ambiguous nucleotides excluded. When converting from FASTA to FASTA+, the minimum read length was specified as 50, so that all shorter sequences were excluded. The FASTA+ format groups identical sequences in the processed FASTA file under a new unique sequence ID (SeqID_#, where # is the number of identical copies), this reduces data size without loss of information [59].

### 2.5. Characterizing HAC1 Splicing Efficiency and Quantifying the Number of Translation Units Mapped to HAC1 Transcripts in S. cerevisiae

Empirical measurement of splicing efficiency (SE) is based on the quantification of spliced and unspliced forms of exonic and intronic sequences. An early attempt to characterize SE is by microarray, which quantifies the exon-exon junction (EE, featuring the spliced form) and exon-intron junction at the 5′ and 3′ sides of an intron (EI5 and EI3 respectively, featuring the unspliced forms) [61]. With the same rationale, a simple but accurate RNA-Seq-based approach to quantify SE has been developed and applied to yeast introns [62]. Here, we apply the RNA-Seq-based approach to quantify *HAC1* SE in *S. cerevisiae*.

To make query sequence files, DAMBE7 [49] was used to extract the *HAC1^u^* sequence from *S. cerevisiae HAC1* gene and three 40nt splice junction sequences were made: EE (20 nt from each exon, overall 40 nt long), EI5 (20 nt at the 3′ end of exon 1 and 20 nt at the 5′ end of intron), and EI3 (20nt at the 3′ end of intron and 20nt at the 5′ end of exon 2) in separate FASTA files. A third query sequence was made which contains the degron sequence: the *HAC1^u^* sequence was truncated to include only the intronic stop codon STOP1 and 20nt upstream of STOP 1 and 17nt downstream of STOP1 (overall 40nt long) and renamed as Target_HAC1u_.

The three 40nt splice junction queries and Target_HAC1u_ were mapped to RNA-Seq and Ribo-Seq BLAST databases using BLAST in ARSDA. The BLAST critical E-value E_i_ where i is the BLAST database is calculated as follows: for i = SRR7265151 with 9699410 sequences of 50 nt each, the effective length for a match of 25 nt is m = 9699410×(50 − 25), and E_i_ of a read of 50 nt matching a query of 40 nt with an exact match of 25 consecutive bases is E_i_ = (40 − 25) × 9699410 × (50 − 25) × 2^(−2 × 25)^ = 3.23 × 10^−6^. To prevent false matches, only mapped reads with match length ≥ 25 and match E-value < critical E_i_ were retained. Hence, the BLAST specifications are as follows: ungapped, minimum match length = 25, E value cutoff = 10^−7^, and Max number of target = 1,000,000. Furthermore, to ensure each reads map over the junction points in our queries, we manually checked each individual mapped region between q.start (beginning of map location on query site) and q.end (end of read map location on query site) to confirm that they overlap the junction points located at sites 20 and 21. All other mapped reads that do not fit these criteria were discarded. 

To determine SE, we quantified the number of RNA-Seq reads mapped to the three splice junction queries. The number of mapped reads on EE junctions (N_EE_) quantifies the spliced mRNA, while the number of reads mapped to EI5 (N_EI5)_ and EI3 (N_EI3_) junctions quantifies two independent measures of the unspliced mRNA. N_EI5_ is typically smaller than N_EI3_ for two reasons [62]. First, step 1 splicing reaction occurs before step 2 splicing reaction, and cleavage of EI5 occurs before EI3 to render less EI5 for sequencing. Second, EI3 is more prone to be included in a library generated with RNA-Seq data because a majority of the RNA-Seq data obtained from the library is enriched in poly(A) tail by oligo-dT. Thus, the total number of *HAC1* mRNA is not measured as the average N_Total_ = N_EE_ + (N_EI5_ + N_EI3_)/2, but is corrected as N_Total_ = N_EE_ + pN_EI5_ + (1 − p)N_EI3_ with proportion p = N_EI5_/(N_EI5_ + N_EI3_), and SE = N_EE_/N_Total_ [41].

Translation efficiency of a given mRNA is correlated with the number of ribosomes involved with the mRNA [63]. Hence, to estimate the number of translation units on *HAC1* transcripts, we quantified ribosome footprints mapped to the spliced *HAC1^i^* transcripts by the number of Ribo-Seq read matches at EE, and we quantified ribosome footprints mapped to the unspliced *HAC1^u^* transcript by the average number of Ribo-Seq reads mapped at EI5, EI3, and TARGET_HAC1u_.

## 3. Results

### 3.1. Efficient HAC1 Splicing Occurs in Unfolded Protein Response (UPR)-Induced S. cerevisiae Cells 

Following an RNA-Seq-based approach to quantify SE, Table 1 shows the number of reads mapped to the three splice junctions in UPR-induced cells. *HAC1^u^* splicing is highly efficient in UPR-induced cells, with SE ranging from 0.955 to 1. N_EE_ ranges from 42 to 140, but when mapped counts were adjusted for differences in Sequence Read Archive (SRA) data size (in number of kilobases in processed datasets), N_EE_/kb becomes more consistent among the four UPR-induced experiments, ranging from 2.7 × 10^−4^ to 4.2 × 10^−4^ (Supplemental File S1). However, in non-UPR cells, only one *HAC1^i^* transcript (N_EE_ = 1, N_EI5_ = 0, and N_EI3_ = 0) was detected in both experiments (SRR7265163, 4) (Supplemental File S1). Consequently, non-UPR results were omitted from Table 1 because SE could not be calculated, and no statements can be made based on the results of a single (or zero) RNA-Seq reads for a gene product.

### 3.2. Translation of HAC1^u^ Is Constitutive in Both UPR-Induced and Non-UPR S. cerevisiae Cells 

Ribosome profiling showed that *HAC1^i^* is translated only in UPR-induced cells, but that a constant and high rate of *HAC1^u^* translation occurs in both UPR-induced and non-UPR cells. Table 2 shows that the number of ribosome protected reads mapped to the EE junction (N_EE_, representing the spliced form) is substantially greater in UPR-induced cells (ranging from 85 to 178) than in non-UPR cells (2 and 5). When adjusted for difference in data size, N_EE_/kb ranges from 2.5 × 10^−4^ to 4.4 × 10^−4^ in UPR-induced experiments and it is 1.4 × 10^−5^ and 6.4 × 10^−6^ in the two non-UPR experiments. This is as expected because *HAC1^u^* splicing is efficient in UPR-induced cells (Table 1). In contrast, the number of translation units mapped to the unspliced *HAC1^u^* transcripts as estimated by TI_HAC1u_ (the average number of ribosome-protected reads mapped to the three unspliced 40 nt queries EI5, EI3, and Target_HAC1u_) are comparable between both cell types (UPR-induced and non-UPR). Surprisingly, TI_HAC1u_ is not substantially lower than N_EE_ in UPR-induced cells (Table 2). Considering all six experiments regardless of UPR, TI_HAC1u_ ranges from 69 to 128. Additionally, when adjusted for differences in data size, TI_HAC1u_/kb ranges from 2.0 × 10^−4^ to 3.4 × 10^−4^ (Supplemental File S1) when all six Ribo-Seq experiments were considered. This range is comparable to N_EE_/kb ranging from 2.5 × 10^−4^ to 4.4 × 10^−4^ in the four UPR-induced experiments.

### 3.3. The Putative Intron-Encoded Degron Is Conserved by Saccharomyces Yeasts but Not by Other Yeasts

Above results showed a constitutive degree of leaky *HAC1^u^* translation in both UPR-induced and non-UPR *S. cerevisiae* cells albeit the presence of a translation block to prevent undesirable *HAC1^u^* translation (Figure 1b). Hence, it would be beneficial if yeast gained an additional post-translational mechanism to rapidly degrade Hac1^u^p. If the previously proposed degron sequence encoded in the last 10 amino acid of *S. cerevisiae* Hac1^u^p [38] is important, then we expect this intronic sequence should be evolutionarily conserved among closely related yeast species.

**Figure 3:**
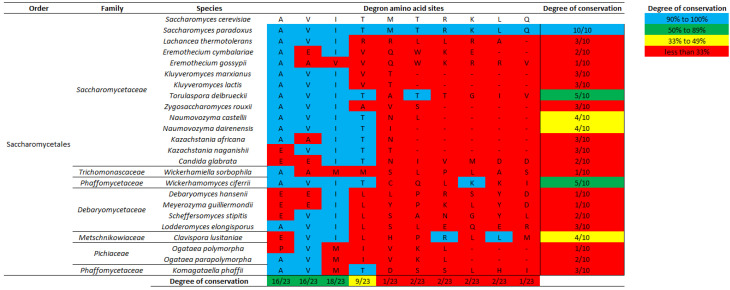
Local amino acid alignments at 24 putative yeast degrons show that the degron sequence is conserved between two *Saccharomyces* species but not by any other yeast (See Materials and Methods for determination of degron-encoded amino acid sequences). Highlighted blue and red are matching and mis-matching amino acid sites, respectively, in yeast degrons against the reference *S. cerevisiae* degron. Degree of conservation designates the total number of matching amino acid residues, at the whole degron (last column) and at each amino acid site (last row), with scores in blue highlights high similarity (90–100%), in green highlights medium similarity (50–89%), in yellow highlights medium-low similarity (33–49%), and in red highlights low similarity (>33%).

Figure 3 shows the local amino acid alignments at putative degrons in 24 yeast species out of the 32 surveyed (See Materials and Methods for degron determination). As expected, we found that the intron-encoded degron is entirely conserved in the two *Saccharomyces* yeast species. Despite this, the degron in *Saccharomyces* yeast is poorly aligned to putative degrons determined in all 22 other yeast species. Two putative degrons that are most similar to that in *S. cerevisiae* are from *Torulaspora delbrueckii* of the Saccharomycetaceae family and *Wickerhamomyces ciferii* of the Phaffomycetaceae family, but both degrons only share 5 out of the 10 amino acid identities with degron of *S. cerevisiae* (Figure 3). Furthermore, three yeasts, *Naumovozyma castellii* and *Naumovozyma dairenensis* of the Saccharomycetaceae family and *Clavispora lusitaniae* of the Metschnikowiaceae family, encode putative degrons that share four out of the ten amino acid identities with degron of *S. cerevisiae*. It is worth noting that the degree of amino acid conservation in all five aforementioned species are mainly attributed to the first four amino acids (A, V, I, and T) which are the most conserved by yeast degrons. Lastly, 17 other yeasts encode putative degrons that share only three or fewer amino acid identities with degron of *S. cerevisiae*. Additionally, we performed a local alignment at degron nucleotide sequences (Supplemental Figure S1), which showed slightly higher overall similarities among degrons and relatively high degrees of conservation at the first 12 nucleotide sites (consistent with the conservation of the first four amino acids A, V, I, and T). To summarize, although the degron sequence in *S. cerevisiae* is poorly conserved by other yeasts at the genus level, the first four degron amino acids (A, V, I, T) are variably highly conserved by yeast species surveyed.

**Figure 4:**
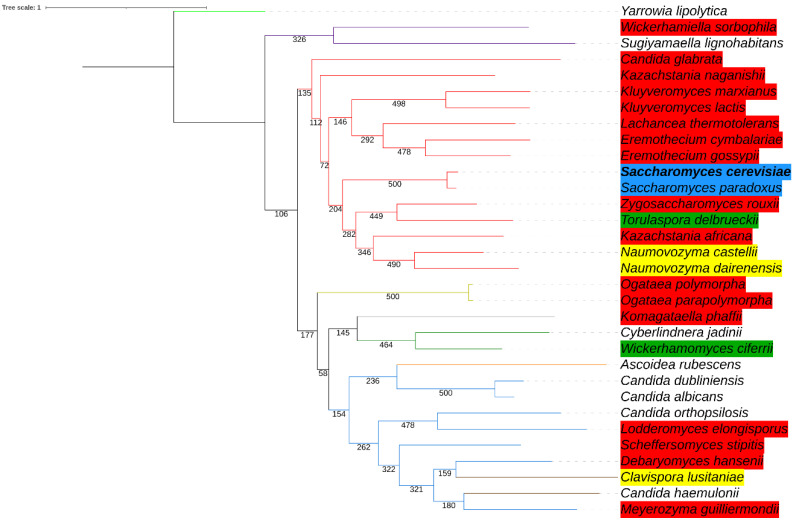
Phylogenetic relationships of 32 yeast species with support values built with whole *HAC1* alignments. The phylogenetic tree is constructed using the maximum-likelihood-based PHYML approach, with best model = GTR + G + I, Bootstrap = 500, and topology re-rooted at *Yarrowia lipolytica*. Yeast species belong to nine families with tree branches highlighted in color: Saccharomycetaceae (red), Trichomonascaceae (purple), Phaffomycetaceae (green), Ascoideaceae (orange), Debaryomycetaceae (blue), Metschnikowiaceae (brown), Pichiaceae (yellow), Phaffomycetaceae (grey), and Dipodascaceae (light green). Color highlighted at species name indicates the degree of amino acid conservation at the whole degron, with reference to the *S. cerevisiae* degron, as shown in Figure 3. Species in which a putative degron cannot be identified are not highlighted by color.

We next reconstructed the phylogenetic relationships (see Materials and Methods) among 32 yeast species to further showcase that the degron sequences are not better conserved by lineages closely related to *Saccharomyces* species. Two phylogenetic trees were reconstructed, one with whole *HAC1* nucleotide sequence alignment (Figure 4) and one with 18S rRNA alignment (Supplemental Figure S2), and both show two major clades representing the Saccharomycetacea family (red) and the Debaryomycetaceae family (blue). While the two trees are not completely identical in topology, they both illustrate that most, but not all, yeast species can be grouped appropriately by their hierarchical taxonomic ranks. For example, *Ascoidea rubescens* of the Ascoideaceae family and *Clavispora lusitaniae* and *Candida haemulonii* of the Pichiaceae family cannot be separated from the clade representing the Debaryomycetaceae family with *HAC1* alignments. Figure 4 additionally shows that species closely related to the two *Saccharomyces* yeasts do not retain better conserved degron sequences (with degree of degron conservation indicated by the same color scheme as shown in Figure 3) than those more distantly related. A putative degron can be determined in all 14 Saccharomycetales, whereas among species of other yeast families, eight out of the 18 do not have an identifiable degron sequence. Nonetheless, most *Saccharomycetales* yeasts have poor similarities when compared to the *S. cerevisiae* degron (Figure 4). Even at the sister group of *Saccharomyces* species, the degron in two species (*Zygosaccharomyces rouxii* and *Kazachstania africana*) are poorly conserved (three shared amino acids) and the degron in the remaining three species (*Naumovozyma castellii*, *Naumovozyma dairenensis*, and *Tourulaspora delbrueckii*) are weakly conserved (four to five shared amino acids). 

**Figure 5:**
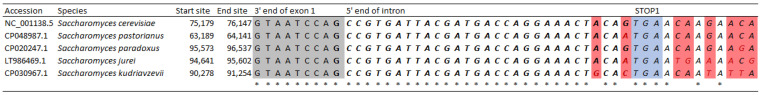
Local sequence alignments shows that the putative degron sequence is well conserved between *Saccharomyces cerevisiae* and four other closely related *Saccharomyces* yeasts. The start and end site respectively denote the start and end locations of the entire *HAC1* gene in the yeast genomes. Shaded grey are the nine sites on the 3′ terminus of exon 1. Sequence in bold is the putative degron sequence in *S. cerevisiae*. Shaded in blue is the intronic STOP1 used by the truncated Hac1^u^p. Shaded in red are non-conserved sites and the * at the bottom denotes fully conserved sites. Nucleotide sequences were aligned using MAFFT G-INS-i.

To further demonstrate that the degron is indeed highly conserved among *Saccharomyces* yeasts beyond the two examined above, we identified and retrieved the degron from the *HAC1* gene of three additional *Saccharomyces* species (*S. pastorianus*, *S. jueri*, and *S. kudriavzevii*). Together, a local nucleotide alignment between five *Saccharomyces* species (Figure 5) at the degrons (encoded amino acids in bold, STOP1 in blue), flanked by nine sites at the 3′ terminus of exon 1 (grey) and nine sites downstream of STOP1, showed that the degron is almost entirely conserved among the five *Saccharomyces* yeasts. The nine sites on the 3′ end of exon 1 are fully conserved among the five *Saccharomyces* species. At the putative degron sequence (bold), only two nucleotide positions are not fully conserved, but all nucleotide differences are synonymous mutations. In comparison, intron sites downstream of STOP1 are poorly conserved, with only three conserved sites out of the nine examined. 

### 3.4. The Degron Sequence Is the Most Conserved Segment at the HAC1 Intron

Above we showed that although the degron in *Saccharomyces* yeast is poorly conserved by other yeast species, the first 4 out of 10 amino acids are relatively highly conserved among putative degrons. To assess the degree of conservation at the degron sequences relative to other *HAC1* gene segments, we next performed comparative sequence analyses using MUSCLE aligned *HAC1* genes of the 32 species listed in Figure 3, with *S. cerevisiae HAC1* gene as reference. Table 3 shows that, by average PhastCons score (See Materials and Methods), the putative intron-encoded degron is well conserved in comparison to the intronic region downstream of STOP1. Furthermore, exon 1 is better conserved than exon 2 and both are better conserved than the intron, but both exons are less conserved than the degron sequence.

**Figure 6:**
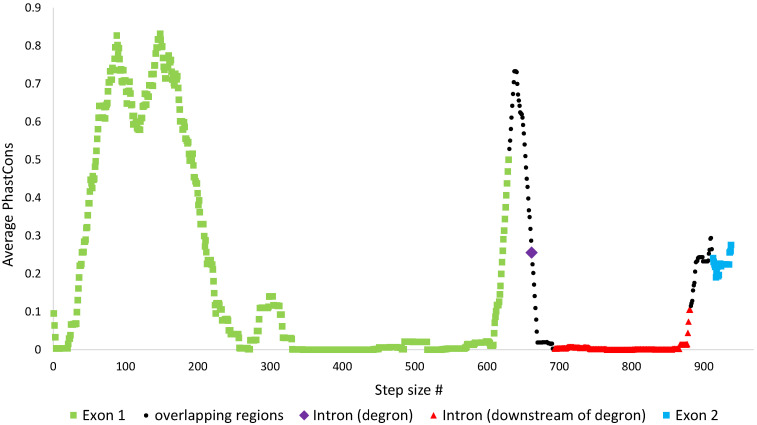
The averaged PhastCons scores for 32 nt segments spanning the entire *S. cerevisiae HAC1* gene, with window size = 32 (length of the degron) and step size = 1. The scores were computed using 32 MUSCLE aligned complete *HAC1* genes and with *S. cerevisiae HAC1* as reference. Scores are color-coded by region, for segments that mapped entirely within exon 1 (green), degron (purple), rest of intron (red), and exon 2 (blue). In addition, average PhastCons scores of 32 nt segments whose sequences overlapped exon-intron junctions or degron-downstream intron sequence are colored in black.

Nonetheless, the above results are not surprising since the length of nucleotide sequence considered at the degron region (32nt including STOP1) was much shorter than that of the other three regions (exon 1, exon 2, downstream intronic region). To better assess the degree of conservation at the degron in comparison to the rest of the *S. cerevisiae HAC1* gene, we computed the average PhastCons scores in short segments, with lengths equaling the length of the *S. cerevisiae* degron (window size = 32), that spanned the entire *S. cerevisiae HAC1* gene (with step size = 1). Figure 6 shows that the degron sequence (purple) is indeed much more conserved in comparison to segments mapping to downstream intronic regions (red). In fact, the degron is comparably conserved against segments mapping to exon 2 (blue). At exon 1 however, segments spanning from sites 42 to 243 (step size # 42 to 212) and 622 to 661 (step size # 622 to 630) are highly conserved with average PhastCons scores higher than that of the degron.

## 4. Discussion

*HAC1* is constitutively transcribed in the *S. cerevisiae* yeast [4,9] and multiple studies have detected spliced *HAC1^i^* mRNAs in both non-UPR [19,44] and UPR-induced cells [19,44,64]. Yet splicing and translation of *HAC1* is tightly controlled and evolution has placed multiple fail-safe mechanism against leaky *HAC1* splicing and translation as these events would allow for the accumulation of Hac1^u^p and Hac1^i^p to induce undesirable UPR [1]. First, Kar2p chaperone proteins hinder Ire1p oligomerization and prevent undesired *HAC1^u^* splicing during non-ER stress [8,42,43]. Second, *HAC1* 5′ UTR interacts with the intron to form a translation block against *HAC1^u^* translation. The Ire1p+Hac1p-mediated UPR signal in *S. cerevisiae* represents a beautiful translation control created by nature. Nonetheless, *HAC1^u^* mRNAs are detected in both non-UPR [38,44] and UPR-induced cells [44,64]. 

In spite of the fail-safe mechanisms mentioned above, a low level of leaky splicing and leaky translation of *HAC1* transcripts may still occur [38]. Thus, a post-translation control would benefit the cell against undesired production of Hac1^i^p and Hac1^u^p. Indeed, Hac1p protein expressions are tightly regulated. For example, while *HAC1^i^* and *HAC1^u^* mRNAs are actively translated during UPR, only Hac1^i^p was readily detected and Hac1^u^p was quickly degraded [4,37,38]. Previous studies proposed that a degradation signal is encoded in the first exon [45], which helps explain why both Hac1^u^p and Hac1^i^p are unstable and rapidly degraded [26,38,46,65,66] and why previous studies [4,26,67,68] were only able to detect Hac1^i^p under UPR conditions. In addition, the budding yeast has been proposed to have evolved a secondary degradation “degron” signal to specifically target and further facilitate the degradation of Hac1^u^p [38]. 

Our investigation employed ribosome profiling and comparative sequence analyses to demonstrate that having an additional intron-encoded degron may indeed be a much-required element to specifically silence undesired Hac1^u^p production. By ribosome profiling, we demonstrated the inability of translation fail-safe to completely inhibit Hac1^u^p production. Both the spliced *HAC1^i^* and unspliced *HAC1^u^* isoforms are constitutively translated in both UPR-induced and non-UPR cells. In fact, Table 2 shows that the translation intensity of *HAC1^u^* is comparable to the translation intensity of *HAC1^i^* in UPR-induced cells regardless of UPR induction. 

Furthermore, through comparative sequence analyses, we determined the intronic sequence encoding the degron to be fully conserved by closely related *Saccharomyces* yeasts (Figure 3 and Figure 5). However, degron conservation is limited to long introns in *Saccharomyces* yeasts and the degron is poorly conserved by other yeast lineages (Figure 3 and Figure 4). Nonetheless, a comparative sequence analysis showed that the first four degron amino acids, A, V, I, and T are highly conserved among putative degrons in 22 non-*Saccharomyces* yeasts (Figure 3), and the degron region is notably more conserved than the downstream intronic regions in yeast species (Figure 6). Similarly, a comparative gene study by Hooks and Griffiths-Jones [25] suggests that the 5′ UTR translation block may also be conserved only in *HAC1* with long introns in *Saccharomyces* yeasts. Future transcriptomics studies on non-*Saccharomyces* yeasts in both UPR-induced and non-UPR conditions may pave the way for determining whether yeasts such as *Candida* species having short *HAC1* introns have acquired alternative mechanisms to regulate UPR and *HAC1* splicing.

**Figure 7:**
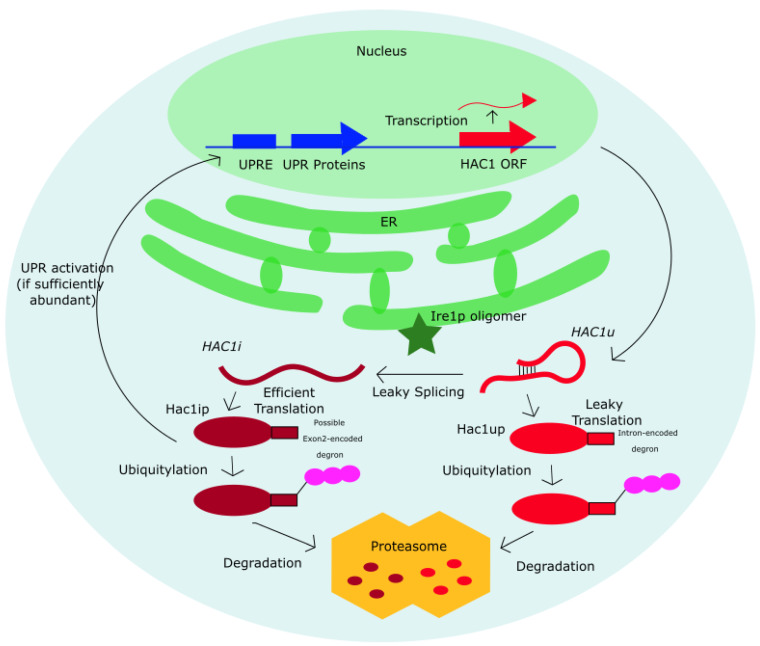
A summary of the proposed independent post-translational control for Hac1^i^p and Hac1^u^p. Both Hac1^u^p and Hac1^i^p may independently encode a degron sequence that signals for their ubiquitylation and degradation, with the former located in the first 10 amino acid of the intronic sequence upstream of the intronic stop codon (STOP1) and the latter located within the 18 amino acid of exon 2.

It is noteworthy that sequence conservation at the degron in *Saccharomyces* yeasts is not a direct evidence of its degradation function and require further verification by application of mutagenesis and proteomics methods. Such experiments have been performed on the *S. cerevisiae* yeast [38], as summarized in the Introduction, but the need for a degron as a “fail-safe” against Hac1^u^p expression remains to be tested in other yeast species. Our findings suggest a functional importance for the degron sequence in *Saccharomyces* yeast to the exclusion of other yeasts, and we expect our findings to motivate future studies to experimentally test the degron function in other *Saccharomyces* yeasts. The evolutionary pressure underlying degron conservation in *Saccharomyces* yeasts suggests a potentially important degron-mediated post-translational regulation to specifically prevent the accumulation of Hac1^u^p, which when unchecked could trigger Ire1p-independent activation of the UPR, since Hac1^u^p is constitutively translated (Table 2) and may be capable of functioning as an active transcription factor at UPRE promoters [38]. Lastly, it is interesting to note that if a degron is present in the last 10-aa of Hac1^u^p, it is also possible that the degradation signal in Hac1^i^p and Hac1^u^p are independently encoded. As visualized in Figure 7, an additional degron could potentially be encoded in the last 18 amino acids of exon 2 for Hac1^i^p.

The homologous *XBP1* gene in humans and other metazoans also contains a conserved intron which undergoes similar Ire1p-mediated unconventional splicing for UPR activation [25]. Similar to *HAC1*, the protein translated from unspliced *XBP1* mRNA, pXBP1(U), is truncated and unable to trigger UPR, while mRNA splicing causes a frame shift to allow pXBP1(S) to encode an effective transcription factor [69]. It has been shown that the C-terminus of pXBP1(U) is sufficient to direct rapid degradation of the protein [70,71], indicating a potential preservation of a degron-like sequence in higher eukaryotes [71].

The causes compelling *Saccharomyces* yeasts to rely on a pair of translational and post-translational regulation mechanisms remain unclear. It is possible that the leniency of the 5′ UTR-intron base-pairing interactions leading to leaky translation imparts an advantage on the cell, by allowing for the proper function of other processes regulating *HAC1* mRNA, such as Ire1p-dependent splicing. On the other hand, it may be that this constitutive production and degradation of Hac1^u^p itself serves a regulatory function, with the intriguing possibility that Hac1^u^p can heterodimerize with residual levels of Hac1^i^p produced by leaky splicing to facilitate its degradation in the absence of ER stress. If Hac1^u^p can function both as a translation factor and regulator of Hac1^i^p, it is possible that the metazoan ortholog of Hac1^u^p, pXBP1(U) [72], can also act as a transcription factor, as pXBP1(U) is known to heterodimerize with its spliced form (pXBP1(S)) to accelerate pXBP1(S) degradation [71]. The Ire1p-Xbp1 pathway in humans may possess unexplored similarities with the Ire1p-Hac1p pathway in yeast, and future comparative research may provide novel insights into the regulation of the UPR in human cells.

## Figures and Tables

**Table 1:** *HAC1^u^* is spliced efficiently in unfolded protein response (UPR)-induced cells. The number of RNA-Seq reads mapped to the three splice junctions were quantified by ungapped BLAST in ARSDA. BLAST databases were made for RNA-Seq datasets from WT yeast cells expressed under DTT-induced UPR (SRR7265165, 66) and TM-induced UPR (SRR7265167, 8) conditions.

SRA Run		N_EE_^1^	N_EI5_^1^	N_EI3_^1^	p ^2^	N_Total_^3^	SE ^4^
UPR-induced (DTT)	SRR7265165	140	1	0	1	141	0.993
	SRR7265166	90	0	0	-	90	1
UPR-induced (TM)	SRR7265167	64	1	0	1	65	0.985
	SRR7265168	42	2	0	1	44	0.955

^1^ N_EE_, N_EI5_, N_EI3_: Number of RNA-Seq reads mapped to exon-exon, 5′ end exon–intron, and 3′ end exon–intron junctions, respectively. ^2^ p: Proportion used to correct bias in N_Total_, equal to p = N_EI5_/(N_EI5_ + N_EI3_). ^3^ N_Total_: total number of *HAC1* transcripts, equal to N_EE_ + p × N_EI5_ + (1 − p) × N_EI3_. ^4^ SE: Splicing efficiency.

**Table 2:** Ribosome profiling shows good *HAC1^u^* translation intensity in both cell types. The number of translation units mapped to the spliced *HAC1^i^* transcripts was estimated by N_EE_, whereas the number of translation units mapped to the unspliced *HAC1^u^* transcripts was estimated by TI_HAC1u_ (the average of N_EI5_, N_EI3_, and N_Target_HAC1u_). Number of ribosome-protected reads mapped to each region was determined by ungapped BLAST in ARSDA. BLAST databases were made from Ribo-Seq datasets of WT yeast cells expressed under non-UPR (SRR7265151, 2), DTT-induced UPR (SRR7265153, 4), and Tm-induced UPR (SRR7265155, 6) conditions.

SRA Run		N_EE_^1^	N_EI5_^1^	N_EI3_^1^	N_Target_HAC1u_^2^	TI_HAC1u_^3^
non-UPR	SRR7265151	5	134	137	126	128
	SRR7265152	2	79	92	98	87
UPR-induced	SRR7265153	178	73	95	126	91
	SRR7265154	107	76	56	79	69
	SRR7265155	113	131	87	134	108
	SRR7265156	85	68	83	129	86

^1^ N_EE_, N_EI5_, N_EI3_: Number of ribosome footprint units mapped to exon-exon, 5′ end exon–intron, and 3′ end exon–intron junctions, respectively. ^2^ N_Target_HAC1u_: Number of ribosome footprint units mapped to the STOP1 query sequence in *HAC1^u^* transcripts. ^3^ TI_HAC1u_: The estimated number of translation units mapped on *HAC1^u^* transcripts, calculated as the average of N_EI5_, N_EI3_, and Target_HAC1u_.

**Table 3:** The averaged nucleotide site conservation at *HAC1* exons and introns (at Degrons and downstream sequences) as scored by PhastCons. The conservation scores at “Complete Alignment” were computed using entire *HAC1* gene alignments, whereas scores at “Separate alignment” were computed using *HAC1* CDS and intron sequence alignments separately.

*HAC1* Gene Region	Average Conservation Score	
	Complete Alignment	Separate Alignment
First Exon	0.207	0.197
Intron (Degron)	0.256	0.308
Intron (Region downstream of Degron)	0.017	0.049
Second Exon	0.235	0.127

## Data Availability

Publicly available datasets were analyzed in this study. This data can be found here: http://dambe.bio.uottawa.ca/Transcriptome.zip (accessed on 1 February 2021) and http://dambe.bio.uottawa.ca/RiboProf.zip (accessed on 1 February 2021). The data presented in this study are available in Supplemental Files S1 and S2.

## Supplementary Materials

The following are available online at https://www.mdpi.com/2076-2607/9/3/620/s1, Supplemental File S1 contain RNA-Seq and Ribo-Seq profiling analyses, sequence annotations and alignments, and computed PhastCons scores. Supplemental File S2 contains Supplemental Figures S1 and S2. The processed RNA-Seq datasets are available online at http://dambe.bio.uottawa.ca/Transcriptome.zip (accessed on 1 February 2021) and the processed Ribo-Seq datasets are available online at http://dambe.bio.uottawa.ca/RiboProf.zip (accessed on 1 February 2021).
