# Supplementary material for: Does Saccharomyces cerevisiae Require Specific Post-Translational Silencing against Leaky Translation of Hac1up?
Source: Microorganisms. 2021 Mar 17;9(3):620. doi: 10.3390/microorganisms9030620 (PMC8002603; doi:10.3390/microorganisms9030620)
Supplement: Supplementary file 1 [file microorganisms-09-00620-s001.zip › Supplemental File S2.docx]

Supplemental Information


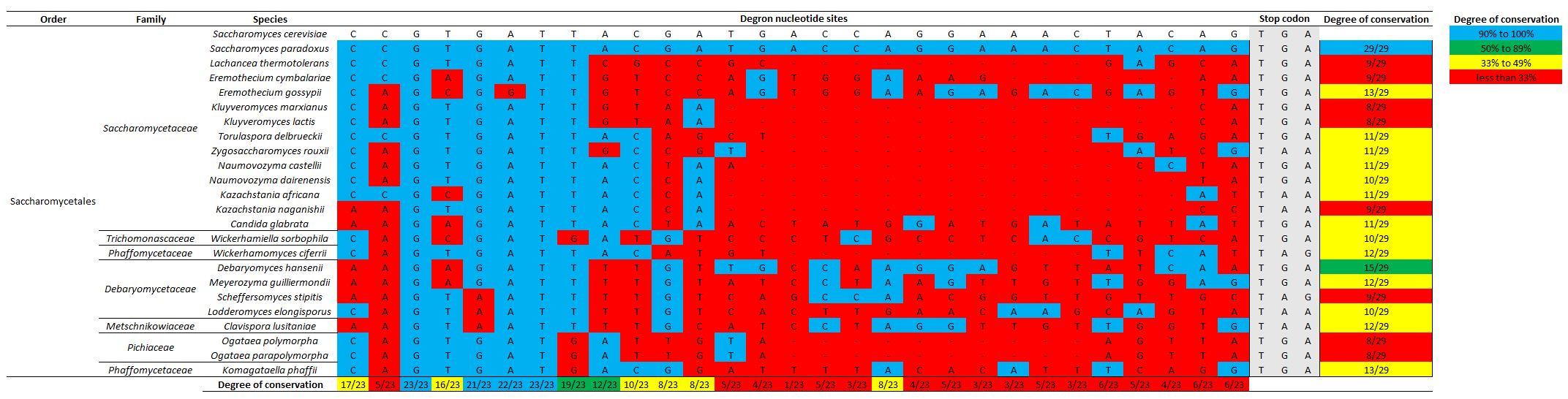


**Figure S1.** Local nucleotide alignments at 24 putative yeast degrons show that the degron sequence is conserved between two *Saccharomyces* species but not by any other yeast. Highlighted blue and red are matching and mis-matching amino acid sites, respectively, in yeast degrons against the reference *S. cerevisiae* degron. Degree of conservation designates the total number of matching nucleotide sites, at the whole degron (last column) and at each nucleotide site (last row), with scores in blue highlights high similarity (90% - 100%), in green highlights medium similarity (50% - 89%), in yellow highlights medium-low similarity (33% - 49%), and in red highlights low similarity (> 33%).


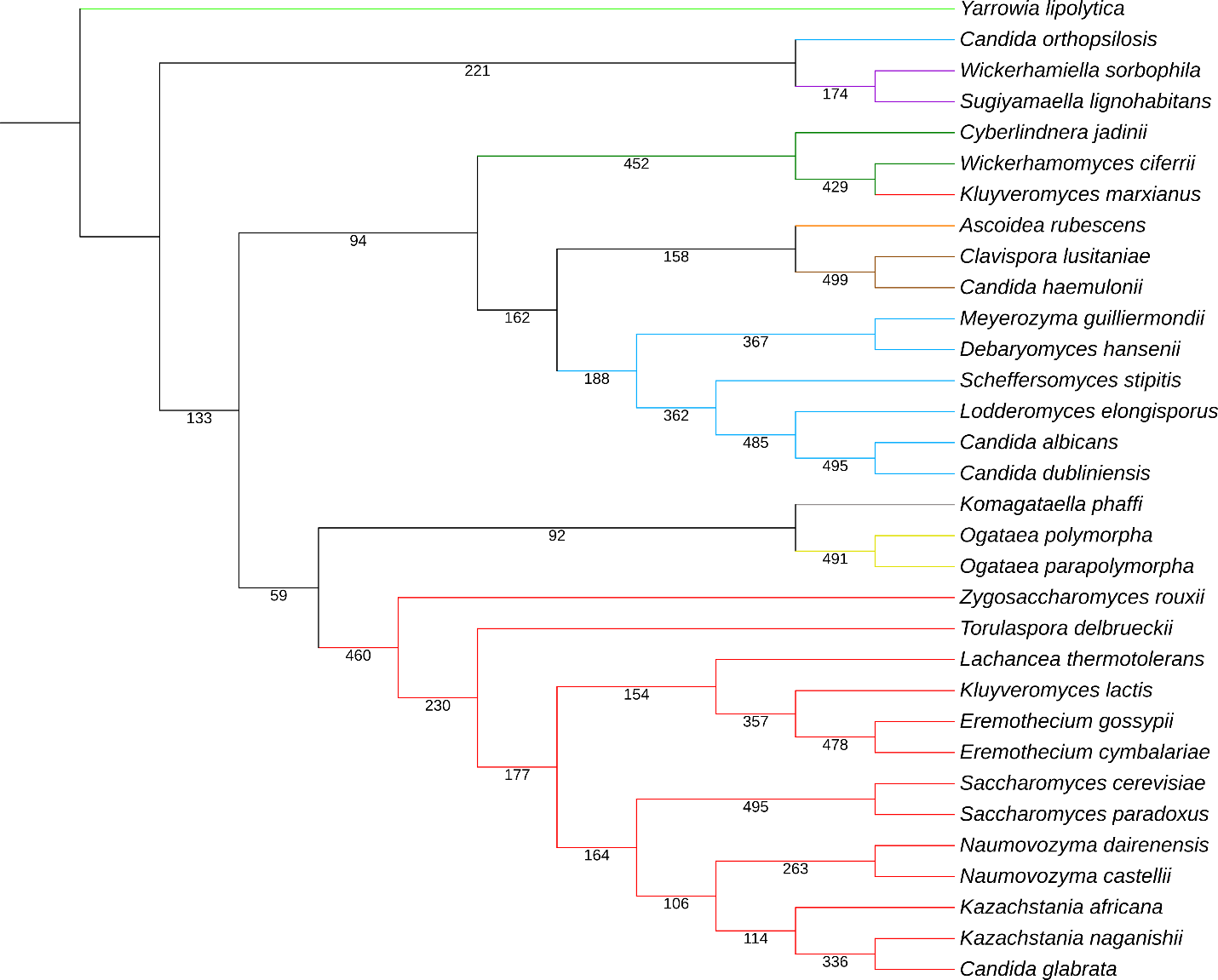
.

**Figure S2.** Phylogenetic relationships of 32 yeast species with support values built with whole 18S rRNA alignments. The phylogenetic tree is constructed using the maximum-likelihood-based PHYML approach, with best model = GTR + G + I, Bootstrap = 500, and topology re-rooted at *Yarrowia lipolytica*. Yeast species belong to nine families with tree branches highlighted in color: *Saccharomycetaceae* (red), *Trichomonascaceae* (purple), *Phaffomycetaceae* (green), *Ascoideaceae* (orange), *Debaryomycetaceae* (blue), *Metschnikowiaceae* (brown), *Pichiaceae* (yellow), *Phaffomycetaceae* (grey), and *Dipodascaceae* (light green).
